# Supplementary material for: TRAECR: A Tool for Preprocessing Positron Emission Tomography Imaging for Statistical Modeling
Source: ArXiv. 2026 Jul 16:arXiv:2511.04458v2. Preprint. [Version 2] (PMC13405421)
Supplement: Supplement 1 [file NIHPP2511.04458v2-supplement-1.pdf]

1 Image Property Quality Control Dashboard: Interpretation & Guidelines

The image property quality control (QC) dashboard was designed to provide summary metrics for MRI and PET NIfTI images. For each uploaded NIfTI image, intensity based metrics were computed from the non-zero foreground region, defined as all non-zero voxels in the image. This foreground region should not be interpreted as brain tissue only. For skull-stripped images, the non-zero region may closely represent brain tissue; however, for non-skull-stripped MRI or PET images, it may also include skull, neck, extracranial tissue, scanner bed, padding, or reconstruction related artifacts.

When DICOM files are available in addition to NIfTI images, the dashboard also provides a *Metadata* tab for structured inspection of DICOM header fields and JSON export of extracted acquisition-level metadata (Figure 1).

1.1 Batch Based QC Strategy

MRI and PET intensity distributions can vary substantially across scanners, imaging sites, acquisition protocols, tracers, reconstruction settings, and preprocessing pipelines. Therefore, absolute thresholds for histogram based metrics such as entropy, skewness, kurtosis, and related intensity features are not expected to be reliable across independent studies or heterogeneous datasets. Instead, the dashboard uses a within-batch robust outlier detection strategy.

Scans were flagged according to the following rule:

- **WARN**: $|z_{\text{robust}}| \geq 3$
- **FAIL**: $|z_{\text{robust}}| \geq 5$

This approach is intended to identify scans that differ substantially from the rest of the uploaded batch. For meaningful interpretation, the uploaded batch should consist of comparable images from the same modality, protocol, tracer, and preprocessing stage. Substantially different image types, such as T1-weighted MRI, PET, masks, different PET tracers, or different preprocessing outputs, should not be combined in the same QC batch because expected protocol differences may be incorrectly flagged as outliers.

The recommended workflow is as follows:

1. Upload a batch of comparable images from the same modality, protocol, tracer, and preprocessing stage.
2. Review the QC heatmap and flagged table.
3. Identify scans that are outliers across one or more metrics.
4. Prioritize scans that show extreme values across multiple metrics.
5. Visually inspect flagged scans using the orthogonal slice preview.

The computed metrics should be interpreted as batch-level QC indicators. The complete per-file QC output, including raw metric values, robust z-scores, and QC flags, is available in the *Full Table* tab, which supports sorting, filtering, and CSV export for offline review or downstream analysis (Figure 2). They can identify scans that differ from the rest of the uploaded batch, but they do not by themselves prove the presence of a specific artifact. Final QC decisions should include visual inspection.

1.2 Interpretation of Histogram-Based Metrics

These metrics are computed from the intensity histogram of the non-zero foreground region.

1.2.1 Entropy

Entropy reflects how spread out the intensity histogram is across intensity bins. Higher entropy can indicate a broader intensity distribution, but it is not specific to one artifact type.

Entropy may be elevated when image noise is increased, when motion, ghosting, or blurring alters the intensity distribution, when intensity scaling or normalization is inconsistent, or when the image contains a mixture of tissues or non-brain foreground structures. Entropy may be very low when the image is near-empty or mostly zero, when heavy thresholding has removed much of the signal, when the image is saturated or heavily clipped, or when the uploaded file is actually a mask, label map, or mostly uniform image.

1.2.2 Skewness

Skewness measures asymmetry of the intensity histogram. Positive skewness, corresponding to a long right tail, may indicate hot voxels, intensity spikes, a small number of very high-intensity voxels, mis-scaling, unusual intensity normalization, or partial field-of-view effects where only high-intensity structures remain.

Negative skewness may indicate unexpected negative values or intensity shifting. However, negative values may also be expected in certain processed images, including z-scored images, harmonized images, residual images, or statistical maps. Therefore, interpretation of skewness depends strongly on the image type and preprocessing stage.

1.2.3 Kurtosis

Kurtosis reflects the heaviness of the histogram tails and the presence of extreme values. High kurtosis often indicates a heavy-tailed intensity distribution, extreme outlier voxels, intensity spikes, or a distribution with many typical voxels and a small number of extreme values. Very low kurtosis can occur when the intensity distribution is unusually flat, when the image has been heavily normalized, smoothed, or transformed, or when the foreground region is overly uniform.

Clipping should be assessed directly using the clipping fraction metric rather than inferred from kurtosis alone.

1.3 Hard QC Checks

In addition to robust outlier detection, the dashboard reports several practical QC checks that can indicate possible processing failures or unexpected image characteristics:

- `NaNInf_Frac`: Fraction of voxels with non-finite values. Any non-finite values are usually indicative of a pipeline failure or invalid image output.
- `Zero_Frac / WHOLE_Coverage`: Extreme zero fraction or abnormal foreground coverage may suggest cropping, padding, incorrect field of view, wrong orientation, or an incorrect file type.
- `clip_frac`: A large fraction of voxels at the maximum intensity value suggests possible saturation, clipping, or rescale problems.
- `WHOLE_NegFrac`: Negative values may be suspicious for PET-like data, although they can be expected in some processed MRI, normalized, residual, harmonized, or statistical images.
- `WHOLE_Nvox`: Very low foreground voxel count may indicate an empty image, failed preprocessing, severe cropping, or an uploaded mask or label file instead of an intensity image.

Overall, the dashboard is intended to flag scans for review. Scans flagged as WARN or FAIL should be visually inspected before making a final QC decision. The dashboard summarizes these interpretation rules, robust-z thresholds, and recommended review steps in the *Interpretation & Guidelines* tab (Figure 3).

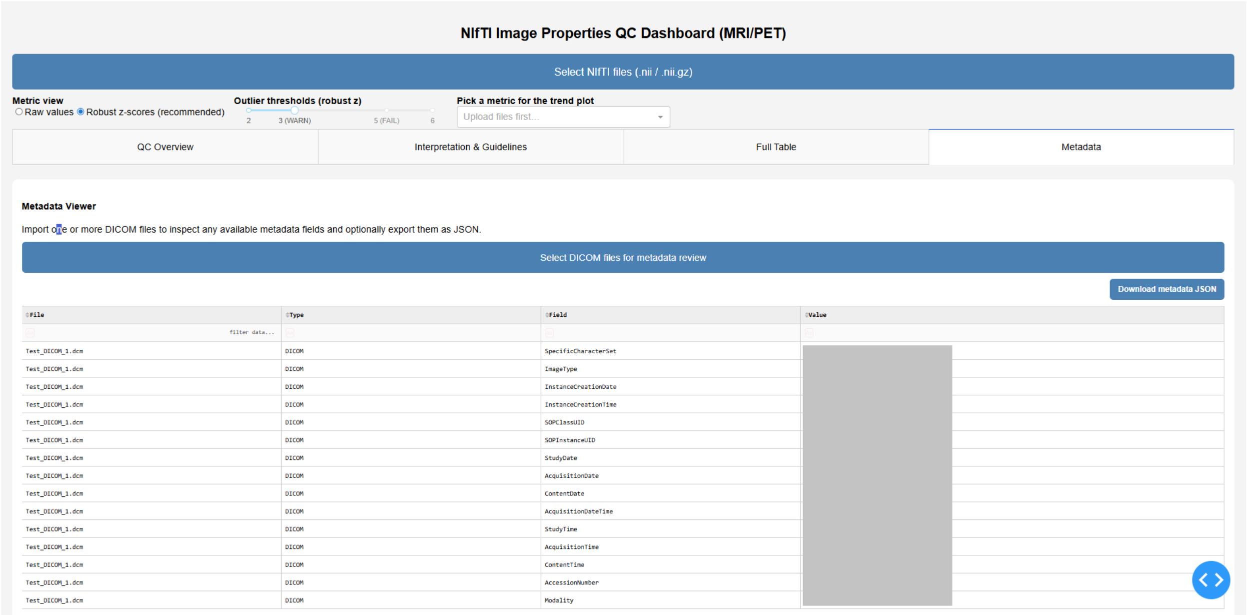

Figure 1: **Metadata tab for DICOM header inspection within the dashboard.** This view allows users to import one or more DICOM files, inspect available metadata fields in a structured tabular format, and export the extracted header information as a JSON file. The tab is intended to support convenient review of acquisition level metadata alongside the image based QC workflow.

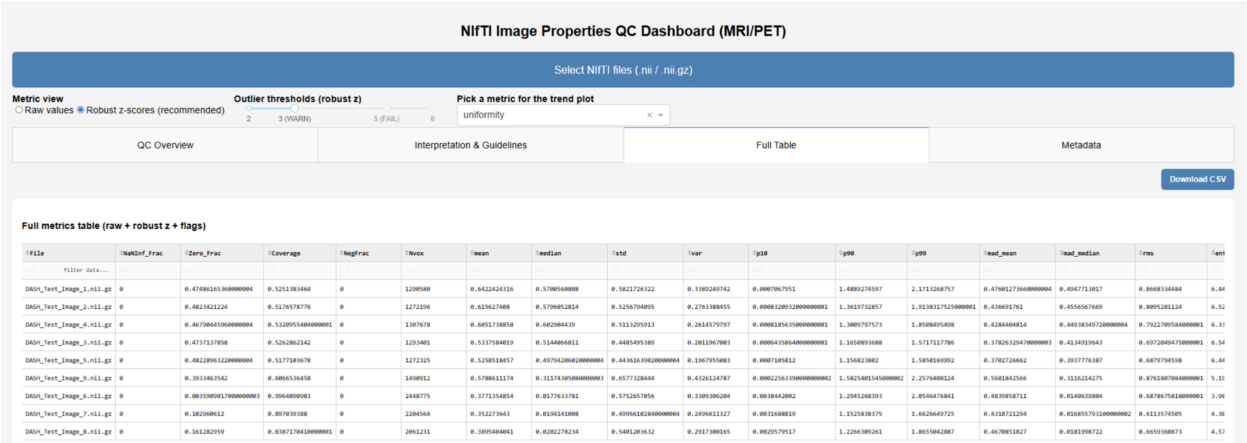

Figure 2: **Full Table tab of the NIfTI Image Properties QC Dashboard.** This view provides a sortable and filterable table containing the complete per file QC output for the uploaded batch, including raw image-property values, corresponding robust z-scores, and QC flags. The *Download CSV* button exports the full table for offline review, record keeping, or downstream analysis.

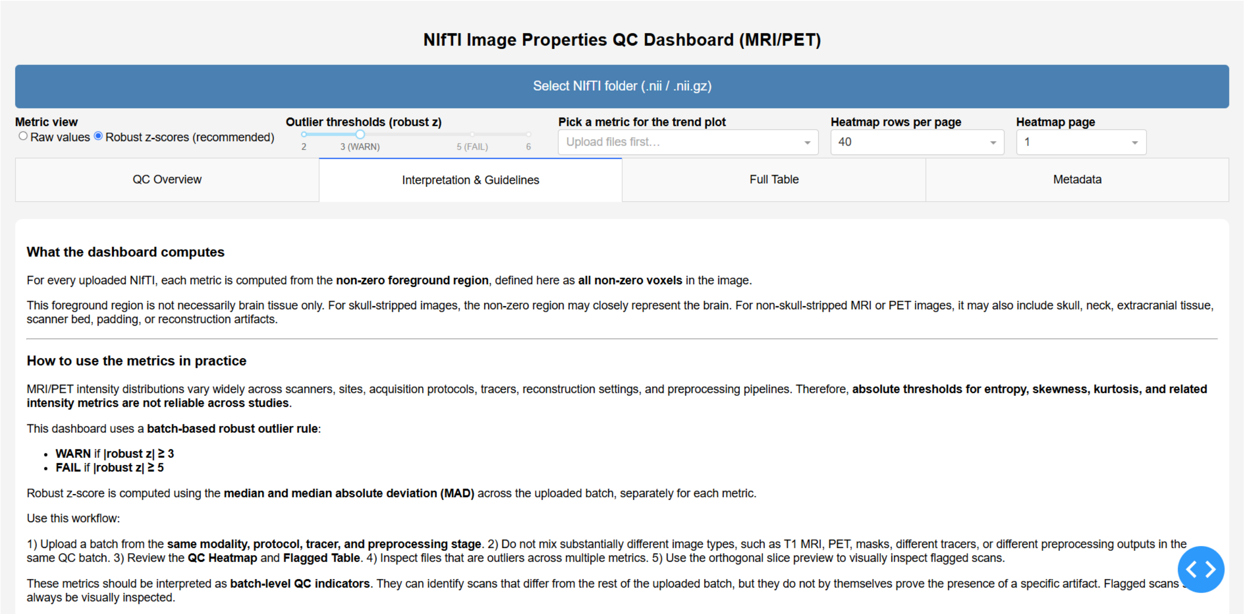

Figure 3: **Interpretation & Guidelines tab of the NIfTI Image Properties QC Dashboard.** This page provides built-in guidance for interpreting the reported image-property metrics and for using the dashboard in practice. It also summarizes the **WARN** and **FAIL** rules based on robust z-scores computed from the batch median and median absolute deviation, outlines a recommended review workflow, and gives practical interpretations of histogram summaries such as entropy, skewness, and kurtosis to support manual follow-up of flagged scans.

2 The Dallas Lifespan Brain Study: Quality Control Review of Wave 3 MRI and PET Images

Image-level quality control was performed for Wave 3 MRI and PET images from The Dallas Lifespan Brain Study that were included in the external validation dataset. The QC dashboard was applied to the available Wave 3 structural MRI, amyloid PET, and tau PET scans. For each modality, image-property metrics were computed across the full batch, and robust z-scores were calculated using the complete set of scans within the reviewed batch. The heatmap visualization was then used to identify scans with unusual metric values, and selected scans were visually inspected using the axial, sagittal, and coronal preview panels. After batch preprocessing, the output visualization interface allowed users to select individual processed cases from a dropdown menu and review the corresponding outputs (Figure 4).

Overall, the reviewed Wave 3 MRI and PET images showed acceptable visual quality for downstream external validation. Most scans did not show obvious artifacts affecting the brain region. Representative examples of normal amyloid PET, structural MRI, and tau PET scans are shown in Figures 5–10. One tau PET scan was flagged by the dashboard because of an extreme image property metric related to the field of view/foreground coverage. Visual inspection showed that the brain region itself was acceptable, and the difference appeared to

be driven primarily by greater inclusion of inferior neck/extracranial anatomy rather than a brain image artifact. Therefore, this scan was retained in the validation dataset.

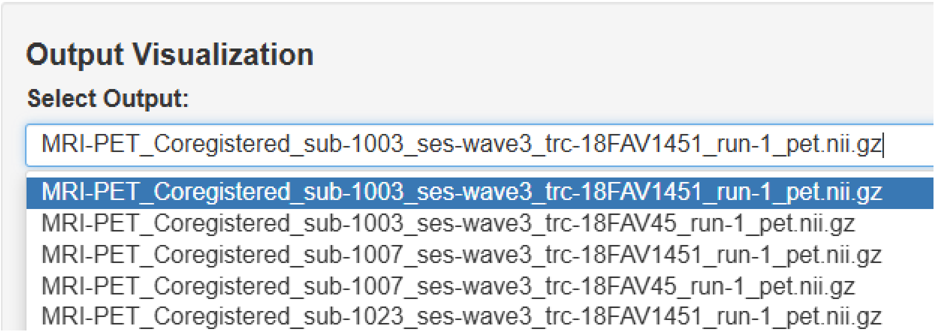

Figure 4: **Output visualization after batch preprocessing** The dropdown menu lists multiple processed cases, allowing users to select and review each case's outputs.

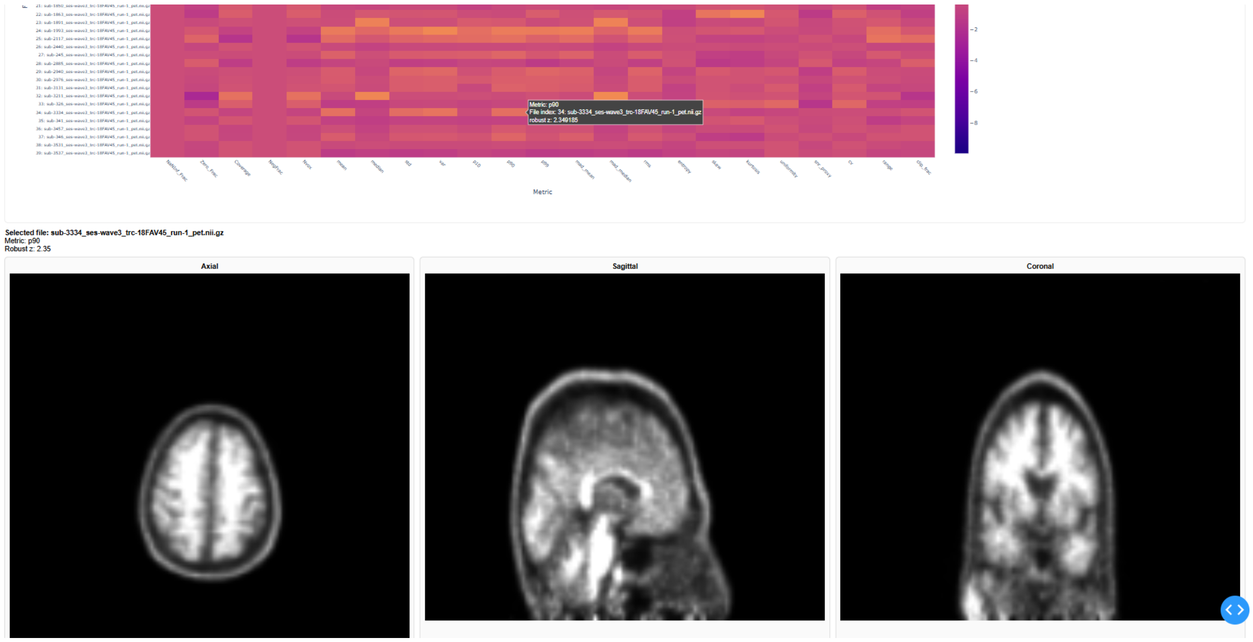

Figure 5: **Representative quality control review of a Wave 3 amyloid PET scan.** The QC heatmap shows robust z-scores for image property metrics, and the orthogonal slice preview shows axial, sagittal, and coronal views of the selected scan. The selected amyloid PET image did not show visible brain image quality concerns.

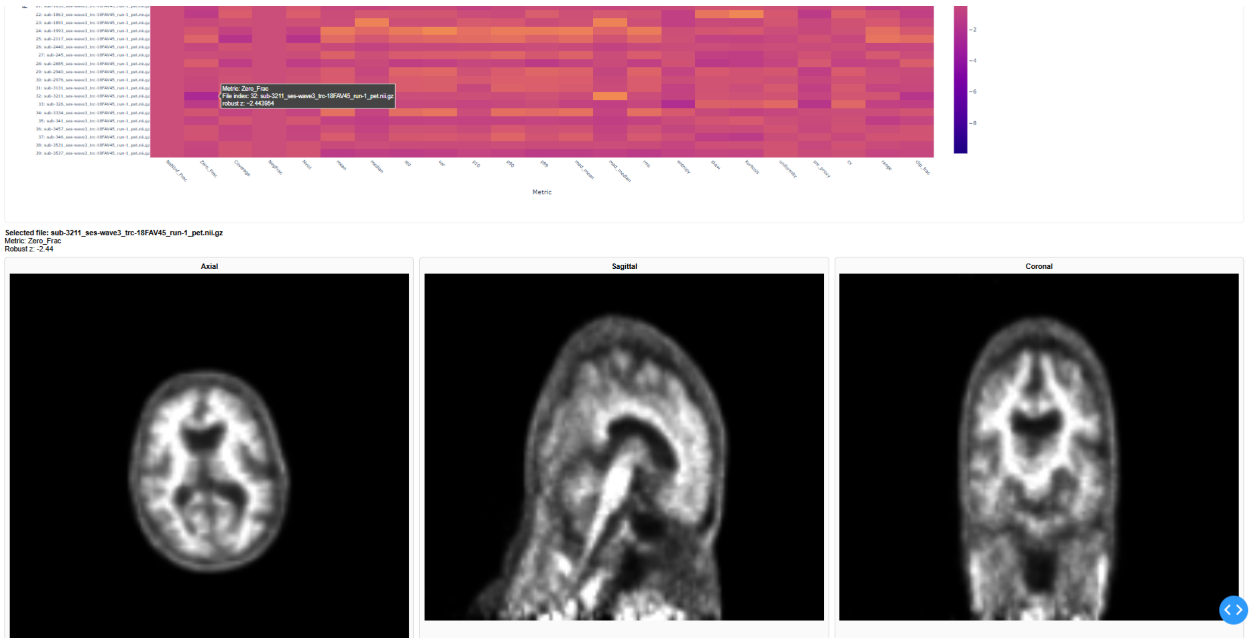

Figure 6: **Additional representative quality control example for a Wave 3 amyloid PET scan.** The selected scan showed expected visual appearance in the orthogonal slice preview, with no obvious artifact affecting the brain region.

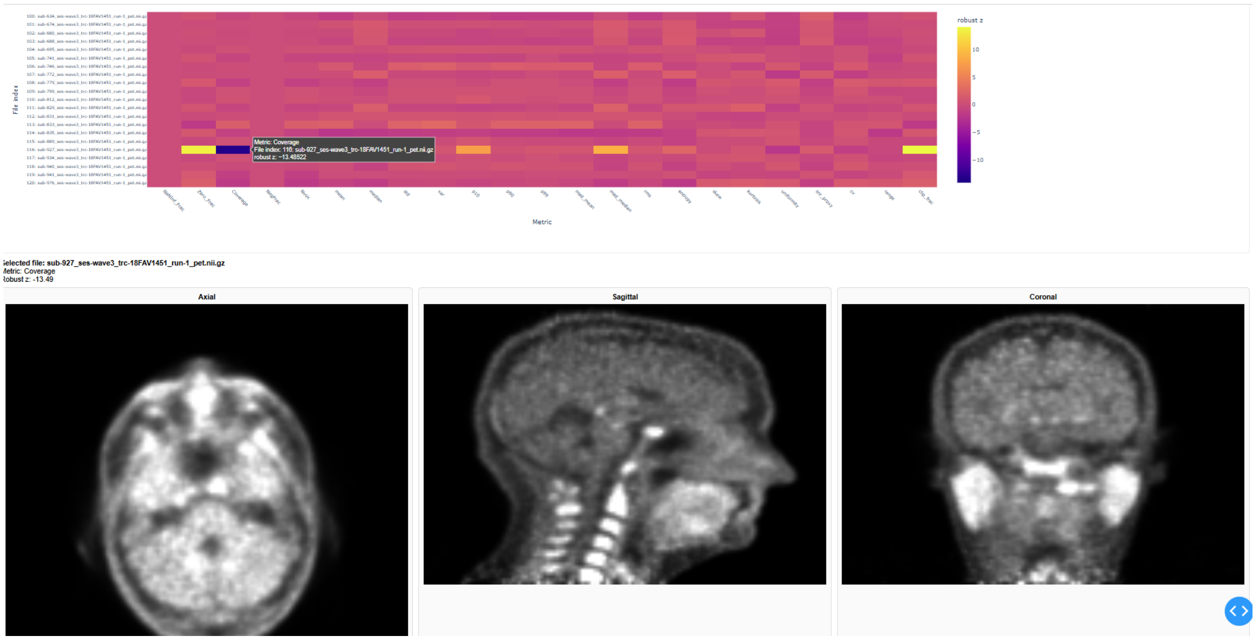

Figure 7: **Quality control review of a flagged Wave 3 tau PET scan.** The scan showed an extreme robust z-score for a coverage related image property metric. Visual inspection indicated that the brain region itself was acceptable and that the flagged metric was primarily driven by greater inclusion of inferior neck/extracranial anatomy rather than a brain image artifact. Based on this review, the scan was retained in the external validation dataset.

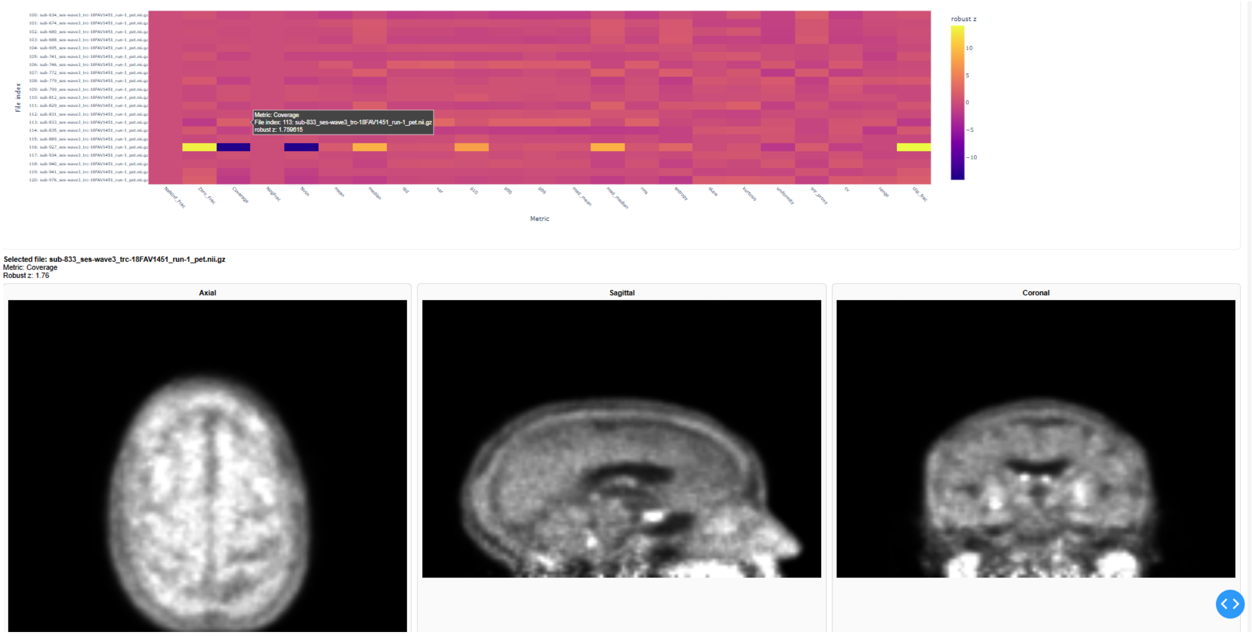

Figure 8: **Additional representative quality control review of a Wave 3 tau PET scan.** The selected scan showed acceptable visual appearance in the axial, sagittal, and coronal views, with no obvious artifact affecting the brain region.

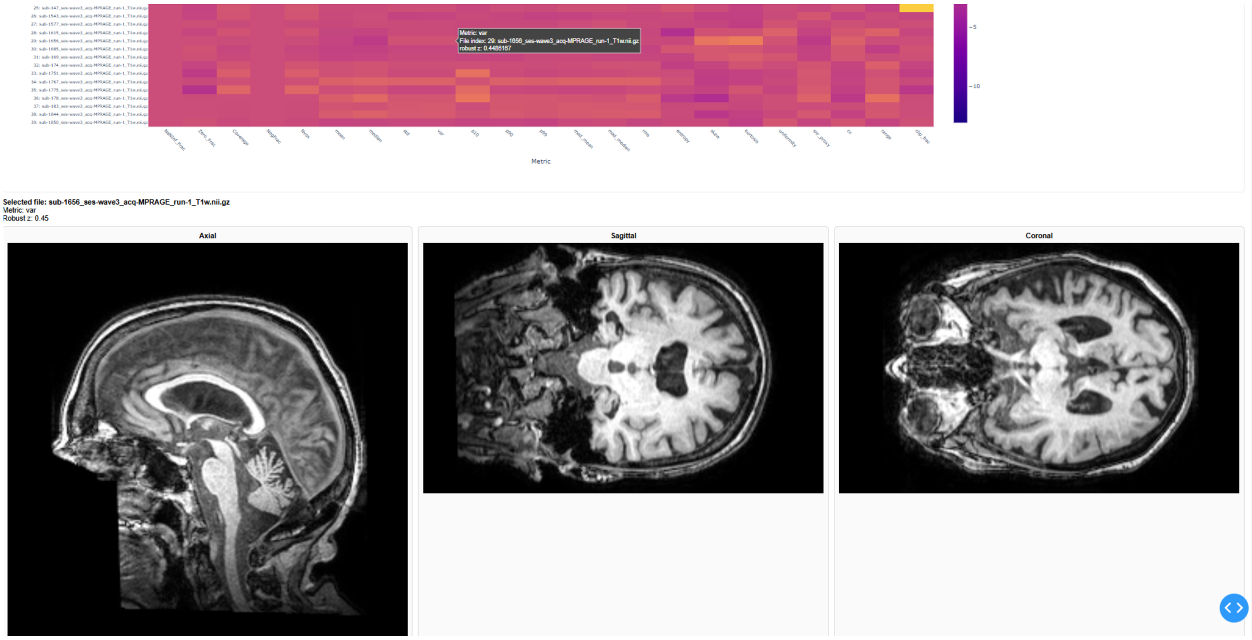

Figure 9: **Representative quality control review of a Wave 3 structural MRI scan.** The dashboard heatmap was used to identify metric level outliers, and the selected MRI was visually reviewed in axial, sagittal, and coronal views. The brain image appeared visually acceptable for downstream processing.

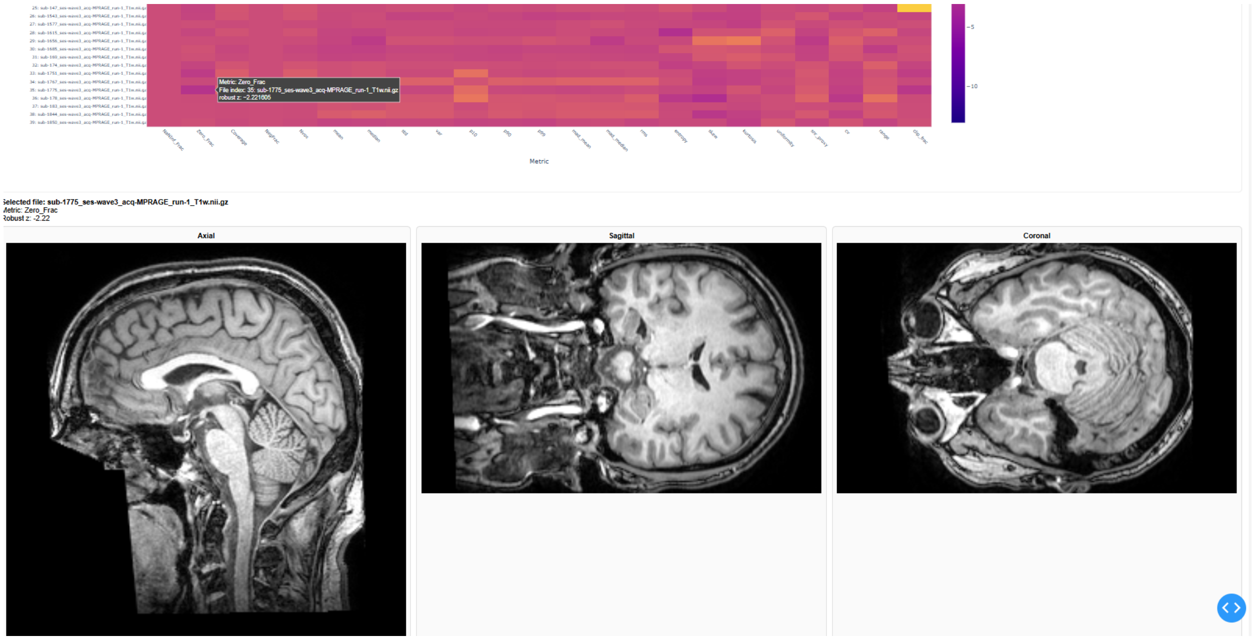

Figure 10: **Additional representative quality control example for a Wave 3 structural MRI scan.** Although image property metrics varied across the batch, the visually inspected MRI showed no visible brain image quality issue in the orthogonal preview and was retained for downstream analysis.

2.1 Post-registration QC of TRAECR outputs

Post-registration QC was also performed on the registered PET outputs generated by the TRAECR workflow. The registered amyloid and tau PET images were evaluated using the QC dashboard to determine whether image property metrics could help identify problematic registrations after pre-processing. The coverage metric was particularly informative: visibly failed registrations often appeared as coverage outliers, whereas visually successful registrations generally remained near the expected batch distribution. For example, an amyloid PET output with visible registration failure showed a coverage robust z-score of -3.12, and a tau PET output with visible registration failure showed a coverage robust z-score of -7.16. In contrast, visually successful amyloid and tau PET registrations showed coverage robust z-scores of 0.21 and 0.48, respectively. These examples indicate that post-registration QC using the dashboard can help prioritize outputs requiring closer review, with visual inspection used as the final confirmation step.

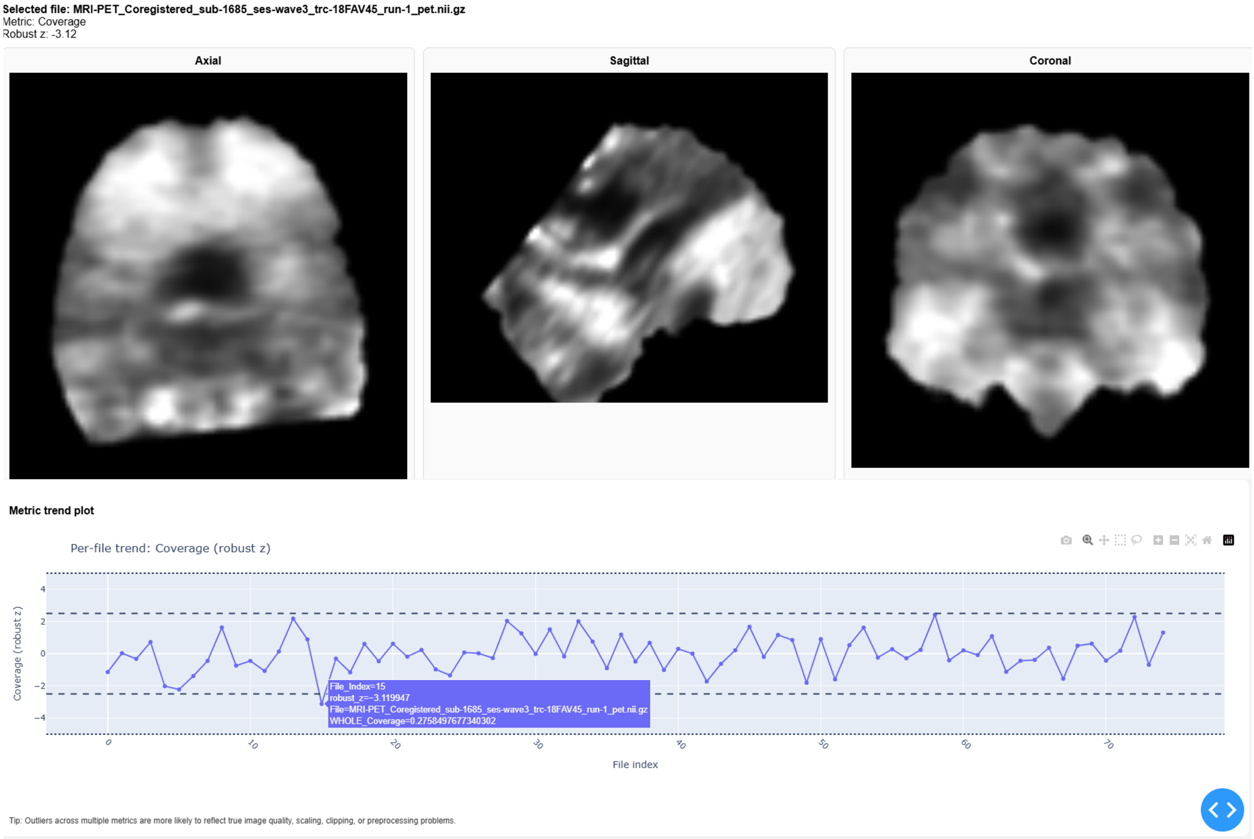

Figure 11: **Post-registration QC example for a failed amyloid PET registration.** The registered amyloid PET output showed visible anatomical misalignment in the orthogonal slice views. The coverage metric was flagged as a WARN-level outlier with a robust z-score of -3.12, indicating abnormal foreground coverage relative to the registered amyloid PET batch.

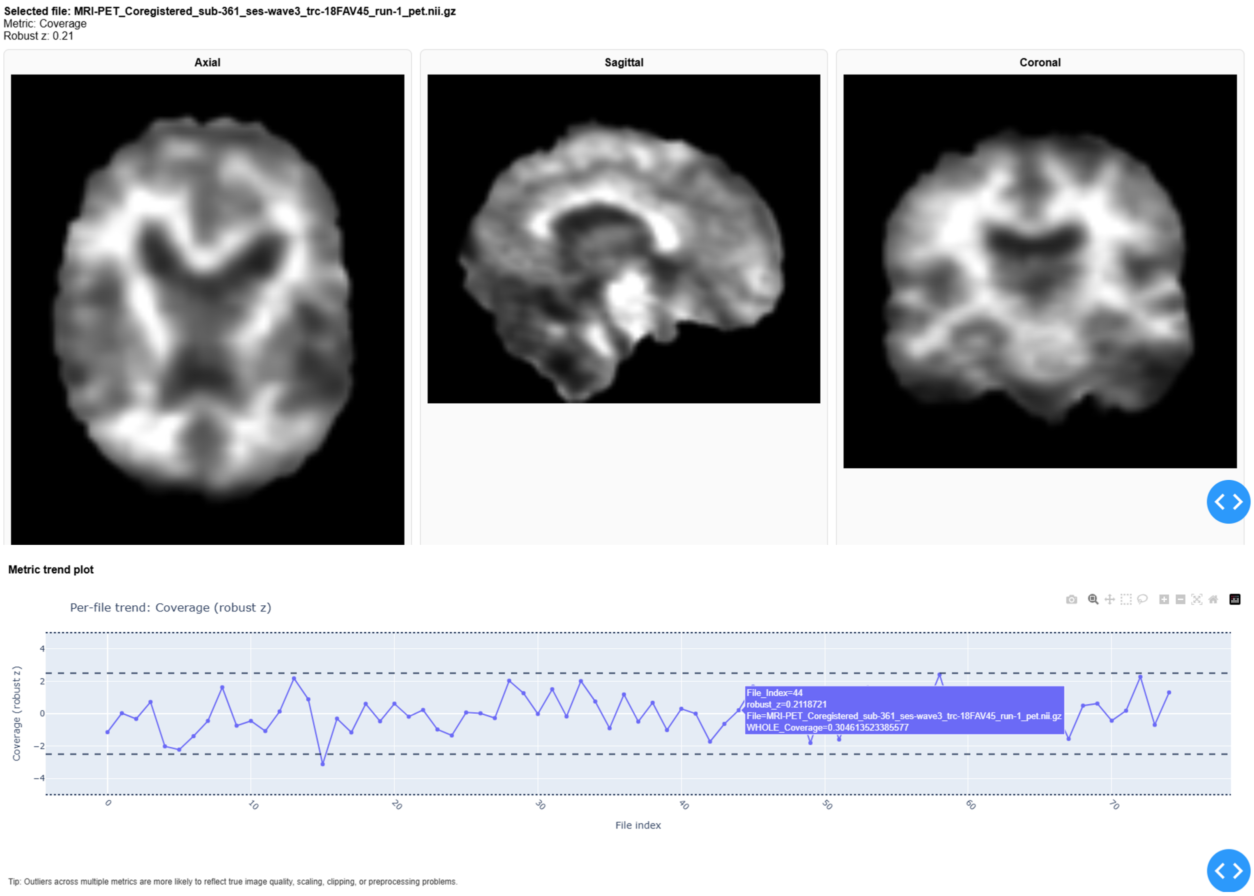

Figure 12: **Post-registration QC example for a successful amyloid PET registration.** The registered amyloid PET output showed acceptable anatomical alignment in the axial, sagittal, and coronal views. The coverage robust z-score was 0.21, remaining within the expected batch range.

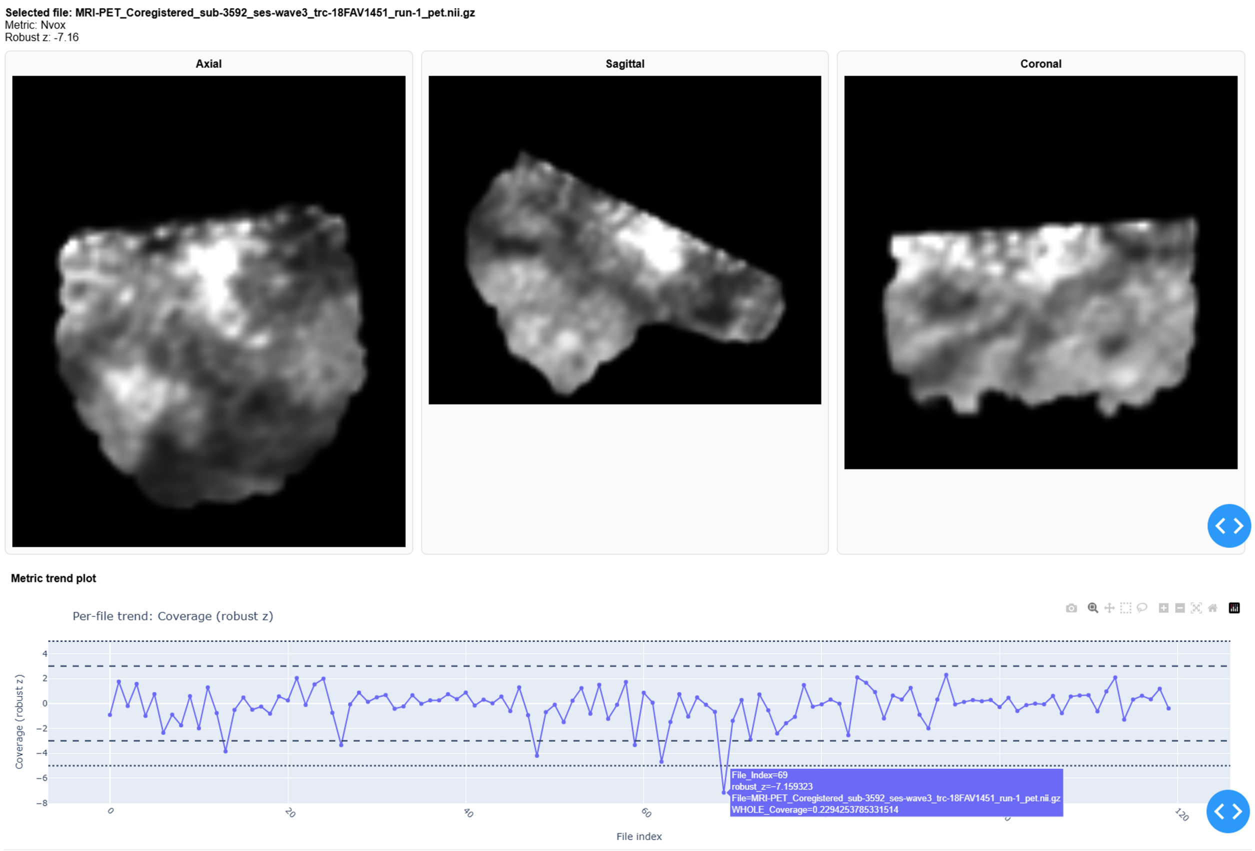

Figure 13: **Post-registration QC example for a failed tau PET registration.** The registered tau PET output showed visible anatomical misalignment in the orthogonal slice views. The coverage metric was flagged as a FAIL-level outlier with a robust z-score of -7.16, indicating markedly abnormal foreground coverage relative to the registered tau PET batch.

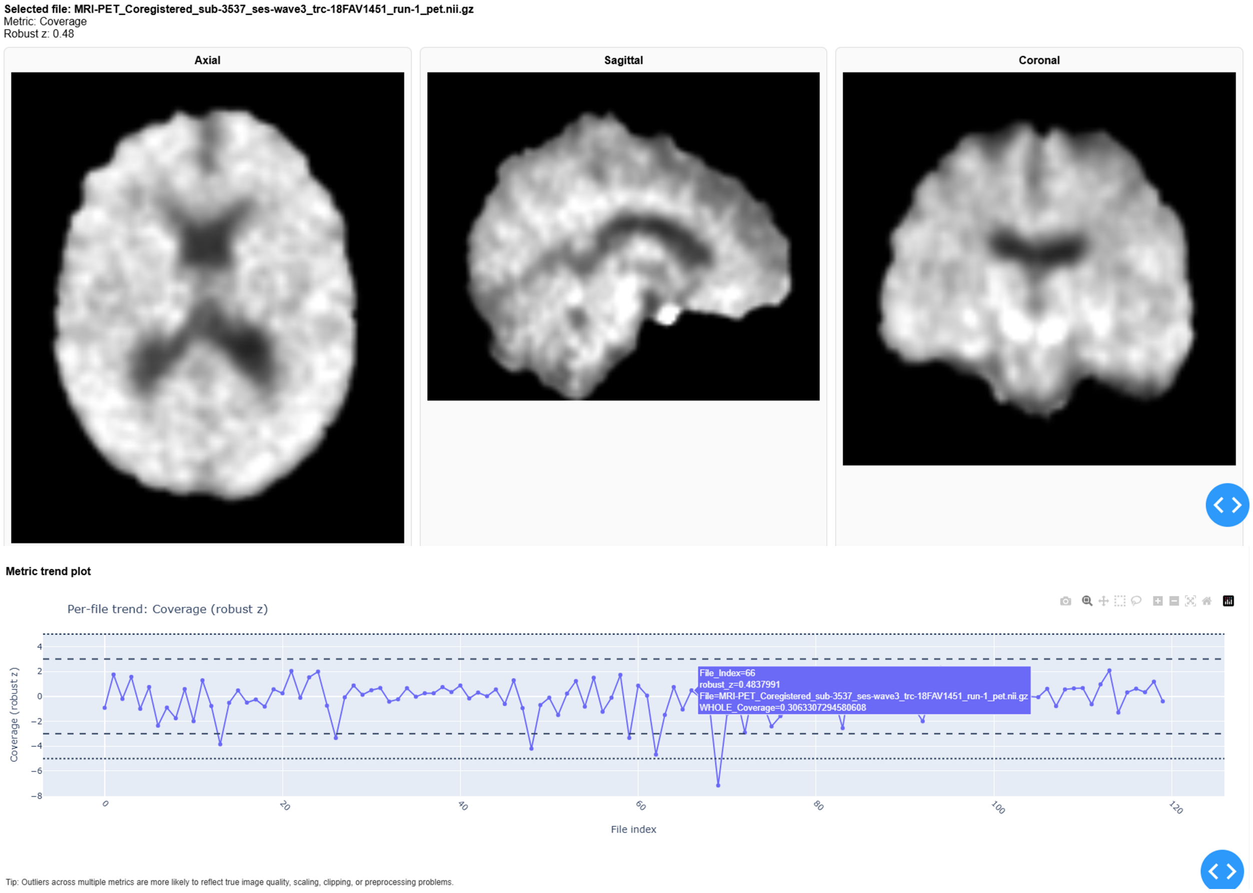

Figure 14: **Post-registration QC example for a successful tau PET registration.** The registered tau PET output showed acceptable anatomical alignment in the axial, sagittal, and coronal views. The coverage robust z-score was 0.48, remaining within the expected batch range.
